# Can Neuropeptide S Be an Indicator for Assessing Anxiety in Psychiatric Disorders?

**DOI:** 10.3389/fpubh.2022.872430

**Published:** 2022-04-26

**Authors:** Agnieszka Markiewicz-Gospodarek, Piotr Kuszta, Jacek Baj, Beata Dobrowolska, Renata Markiewicz

**Affiliations:** ^1^Department of Human Anatomy, Medical University of Lublin, Lublin, Poland; ^2^Students Scientific Association at the Department of Human Anatomy, Medical University of Lublin, Lublin, Poland; ^3^Department of Holistic Care and Management in Nursing, Medical University of Lublin, Lublin, Poland; ^4^Department of Neurology, Neurological and Psychiatric Nursing, Medical University of Lublin, Lublin, Poland

**Keywords:** psychiatry, NPS, mental disorders, psychiatric complications, neurobiology, psychiatry

## Abstract

Neuropeptide S (NPS) is a neuropeptide primarily produced within three brainstem regions including locus coeruleus, trigeminal nerve nucleus, and lateral parabrachial nucleus. NPS is involved in the central regulation of stress, fear, and cognitive integration. NPS is a mediator of behavior, seeking food, and the proliferation of new adipocytes in the setting of obesity. So far, current research of NPS is only limited to animal models; data regarding its functions in humans is still scarce. Animal studies showed that anxiety and appetite might be suppressed by the action of NPS. The discovery of this neuromodulator peptide is effective considering its strong anxiolytic action, which has the potential to be an interesting therapeutic option in treating neuropsychiatric disorders. In this article, we aimed to analyze the pharmaceutical properties of NPS as well as its influence on several neurophysiological aspects—modulation of behavior, association with obesity, as well as its potential application in rehabilitation and treatment of psychiatric disorders.

## Introduction

Most of us have negative associations with stress, although we can distinguish distress i.e., “bad stress” which is dangerous to health especially when it is very tense and frequent, and eustress i.e., “positive stress” leading to satisfaction without negative effects of its impact. It functions as a reaction to specific and general stimuli, which are commonly called stressors. Each stressor elicits unpleasant experiences, whose effect is discomfort, both physiological and psychological. A stressful situation in which an individual can find themselves leads to the activation of all possible systems with the goal of alleviating the uncomfortable situation.

The mobilization of an organism, as well as its adaptation, is dependent on many factors, among other personal and environmental conditions, as well as experiences accumulated over the course of one's life (1). The standard definition of stress divides it into two types, motivating or demotivating (2). Whereas, the effect of the former can lead to a positive outcome, a person activates one's own resources to act or for defense, the latter causes a negative effect, eliciting unpleasant and unwanted reactions, in which a person can lose control over rational behavior.

The model of cascading, chronologically linked reactions of an organism to stress has been long known. Most of these reactions involve the activation of various systems: neurohormonal, neuromodulating, and neurotransmission, all incorporated into the HPA (hypothalamic-pituitary-adrenocortical) axis (3).

The key goal of the cooperation of these systems is the restoration of balance, or homeostasis, to the organism. Of significant importance in this process are the recently discovered neuromodulating peptides, or neuropeptides, specifically neuropeptide S, as well as orexin (OX), nesfatin-1, spexin (SPX), and relaxin-3 (RLN-3) (4).

Considering that neuropeptide S (NPS) shows anxiolytic effects, it's worth closer examining its mechanism of function, as well as the possibilities of its use as a marker for levels of fear, anxiety and cognitive deficits, or those symptoms which most commonly dominate in persons with psychiatric disorders (5).

The analysis and verification of the thematic publications was based on the results of the research to date and a comprehensive review of PubMed and Scopus (as of 12/12/2021). Each article with the following key words likes neuropeptide-S, mental disorders, stress was included in the analysis. The use of keywords allowed for the narrowing of the information related to the analysis of the NPS level in mental disorders, which is the starting point for this publication. Since the undertaken topis are an innovative research issue, the scope of the current literature is not wide. Nevertheless, the authors took up this topic bearing in mind the importance of this neuropeptide in the stress regulatory system. The publication contains thematic subsections analyzing the impact of stress on the body's reactions, the level of neuropeptide-S and mental disorders.

## The Mechanism of Stress Reaction

In response to stress reaction takes place in the nervous system, which reacts on stressogenic stimuli, both internal and external (6). Their reception and analysis are based on the activation of various interconnected structures of the nervous system. Their communication permits a cascade of biochemical changes, which result in the modulation of the levels of neurotransmitters (acetylcholine/Ach, adrenaline/A, noradrenaline/NA, dopamine/DA, glutamate, GABA, serotonin/5-HT) (1, 7), as well as the levels of neuropeptides (oxytocin/OXT, OX, nesfatin-1, SPX, NPS, RLN-3) (8–16). The effect of the mutual modulation of these neuro-markers are two types of excitement. The first type of excitement consists of the activation of the sympathetic-adrenomedullary (SAM) system, which causes the triggering of immediate responses in a stressful situation, while the second activates the hypothalamic-pituitary-adrenocortical (HPA) axis, which triggers time-delayed responses. Both loops activated in a stressful situation work synergistically, and their basic goal is the return of homeostasis in an organism. The activation of the SAM system incorporates the following: cerebral cortex, thalamus, and amygdala. The signals promulgated through this circuit consist of basic information, which are the primary response to stress (17). The secondary response creates an integrated message, which is mainly analyzed by the hippocampus, and in which the frontal, temporal, parietal, and occipital cortex areas are engaged (18). The integration of specific regions of the brain enables the analysis of the entirety of the information which an individual in a stressful situation receives and are the effect of behavior.

## Anatomical Distribution of NPS and Its Biochemical Properties

There are currently around one hundred neuropeptides that have been identified. Most are grouped into clusters depending on structure and biochemical function. Among these numerous neuropeptides we focus on the following peptides: neuroenteric, opioid, hypothalamic, and pituitary (18).

**Figure 1:**
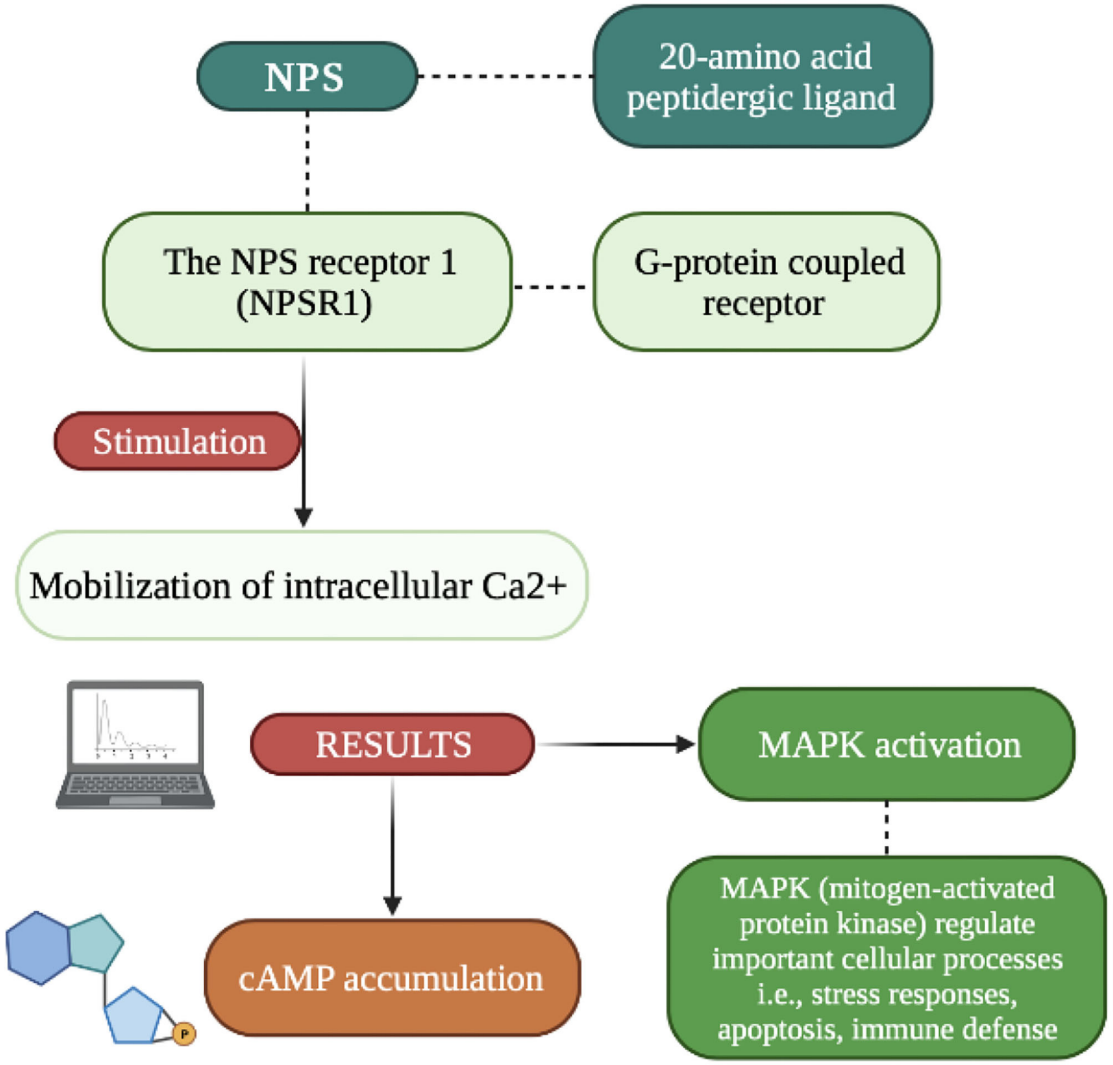
Picture showing the effects of NPSR1 receptor stimulation (82, 83).

Recently discovered, and very interesting, is neuropeptide S (NPS), which is formed by twenty amino acid residues. It is part of the excitatory signaling system which under the influence of changes in the action potential in the lower part of the axon causes a cascade of biochemical changes. The effect of these changes in the release (under the influence of Ca^2+^) by exocytosis of neuropeptides accumulated in the vesicles (18). While the low frequency of stimulation causes the rapid release of classic neurotransmitters, increasing the stimulation results in the co-transmission of both mediators. It should be remembered that the release of the dominant inhibitory neuropeptide inhibits the action of classical transmitters. Since NPS acts as a local neuronal mediator, its effect is like that of hormones, it is temporarily prolonged (18). Its main responsibilities are signaling functions and modulation of various states, such as wakefulness, fear, or mood. The NPS release mechanism involves pathways Gαs and Gαq (inter-receptor communication) aimed at increasing intracellular cAMP and releasing Ca^2+^ from the endoplasmic reticulum (19). NPS activates the orphan protein G-coupled receptor (NPSR1) which is responsible for the increase in intracellular Ca^2+^ and cAMP, and acts as an excitatory messenger. NPS peptide precursor mRNA is found in limited regions of the brain in contest to NSPR1 mRNA which was found thought the CNS (Central Nervous System). The NPS/NPR1 system plays an important role (as shown in Figure 1) in stress reactivity, HPA axis activation and has also been attributed great importance in the neurobiology of sleep and wakefulness, modifications related to cognitive functions (memory consolidation, concept generalization) and personality expression (4, 13–15).

The expression of NPS in the brain is uneven. The studies on rat and mice revealed that the NPS precursors mRNA is strongly expressed in hypothalamus, thalamus, hippocampal formation, and para-hippocampal regions (20). NPS-expressing neurons are presented most abundantly within the cell populations located close to the locus coeruleus, and sensory and caudate nuclei (13). Kölliker-Fuse nuclei and lateral parabrachial nuclei constitute the most important source of NPS in vertebrate brain (21). Besides, NPS-expressing neurons were reported to be found in the amygdala, thalamus, hypothalamus, as well as frontal and hippocampal areas, however in significantly smaller number compared to the aforementioned locations.

The target of NPS is the NPSR receptor which is located mainly in the limbic structures (4, 18), mainly responsible for various responses, such as emotional, those associated with hunger, thirst, or sexual drive. These conclusions are confirmed by Reinscheid and Xu who claim that the engagement of NPS and NPSR involve emotional behavior and are visibly associated with HPA axis activity (16). This relationship is supported by the authors' study, which demonstrates and increase in plasma concentrations of ACTH (adrenocorticotropin-releasing hormone), corticosterone, as well as increased expression of CRF (corticotropin-releasing factor) and AVP (arginine-vasopressin peptide).

Both neuropeptides and neurotransmitters are involved in the complex function of transmitting nerve impulses between neurons. Neurotransmission is thus a constantly observed electrical phenomenon that is present in both the central and peripheral nervous systems. This intercellular transmission of information occurs via synapses, which are connections between the axon of one cell with the axon, dendrite, or body of another cell (2). The propagation of impulses causes activation of receptors and stimulation of target enzymes. These processes involve many mediators that interact with each other, such as neurotransmitters, neuropeptides, hormones, and cytokines (22). The site of neuropeptide synthesis is the endoplasmic reticulum and Golgi apparatus, in which neuropeptides are accumulated in the so-called “large vesicles”/LDCV (large dense-core vesicles) through numerous branches diverging from it. The neuropeptides accumulated in the vesicles are transported taxonomically to the nerve endings, where the change in action potential causes their release. The site of neurotransmitter synthesis is the cytosol (cytoplasm) where small molecule neurotransmitters are produced in the so-called “small vesicles”/synaptic vesicles (11, 16, 18). Both classical neurotransmitters (neurotransmitters) and neuropeptides are stored in presynaptic nerve endings and act mainly as chemical mediators with neuropeptides acting especially as so-called local modulators.

The basic difference between these transmitters is that neuropeptides have short amino acid chains, resulting in slow activity, prolonged response, and a site of action away from the site of release. Moreover, their effect is more potent, resulting in an apparent change in the modulation of the regulatory mechanism of metabolic pathways as well as gene expression (18). Neuropeptides also could interact with other receptor proteins and, importantly, do not undergo reuptake (2, 4).

In contrast to neuropeptides, classical neurotransmitters (acetylcholine, glutamate, GABA, epinephrine, norepinephrine, serotonin, dopamine) act rapidly and bind to only one specific receptor. Neuropeptides that act slowly and require stronger stimulation have a broad spectrum of activity that allows them to bind to other neuropeptides receptors belonging to the family of G protein-coupled receptors (e.g., oxytocin receptor, AVP receptor, galanin receptor) (18, 23). The difference between mediators emphasizes (especially in the case of NPS) how important is the participation of neuropeptides in the change of protein expression. The glutamatergic system is responsible for HPA axis regulation, synaptic modulation, and neuronal plasticity.

**Figure 2:**
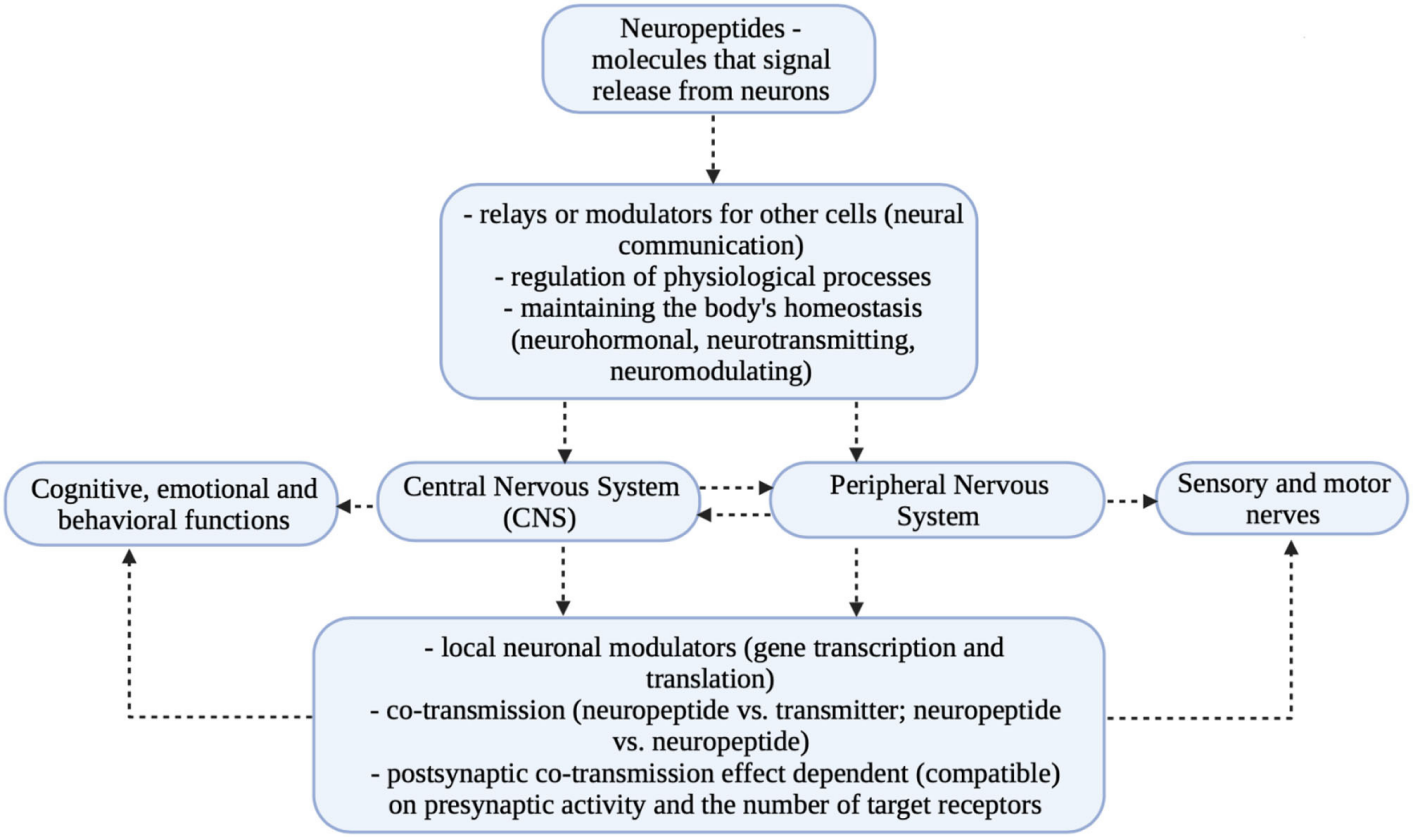
Multifaceted action of neuropeptides (18, 22–25).

Studies in recent years have shown that affective disorders, which are accompanied by multiple stress reactions, cause visible dysregulation in neurotransmitter levels and changes in CNS structures. The observed deficits concern primarily the reduction of the number of cells and weakening of neurogenesis in the hippocampus and in part of the frontal lobe (prefrontal region) (24, 25). The described relationships are presented in Figure 2.

Previous animal studies show that the rat locus coeruleus (LC) NPS is not co-localized with noradrenaline in the actual LC but is in neighboring neurons. Studies in transgenic mice have shown that the total number of NPS neurons in the brain is ~500 (26). Immunohistochemical analyzes showed the highest densities in the hypothalamus, thalamus, and amygdala structures (26, 27). Increased NPS release under stress was revealed in the rat amygdala using the micro-dialysis method (26). Moreover, with sleep deprivation the regulation of NPS transcripts was observed (28). As in rodents, clusters of NPS neurons have been demonstrated in the human lateral brachial nucleus and in the perirenal region, although at a much different level than in rodents. A large group of NPS neurons identified in the central gray matter of the bridge. The analyzes revealed around 22 000 NPS-producing neurons.

## NPS As A Modulator of the HPA Axis

The HPA axis, also known as the stress axis, operates with some delay (up to 30 min) (4). The delay effect causes the body to have the opportunity to prepare for, control, and time the resolution of a difficult situation. Since the main action of NPS is the promotion of locomotor activity, behavioral arousal, wakefulness and monitoring of anxiety levels, we can with some certainty assume that NPS may be an initiator of specific behavior (29) related to the influence of the HPA axis (hypothalamic-pituitary-adrenal) on the course of the stress response (30, 31). This assumption is supported by some researchers, including Smith et al. who demonstrated that *in vivo* administration of NPS causes activation of the HPA axis and thus the release of corticotropic hormone (CRH), adrenocorticotropic hormone (ACTH) and corticosterone (32). Other studies (animal model), on the other hand, demonstrate the opposite situation, in which it is CRH that induces NPS release, mainly in the amygdala (33). Both examples undoubtedly emphasize the high dependency between the mutual correlation of hormones induced by the stressful situation. The conclusion of the cited results undoubtedly confirms the complexity of biochemical transformations whose starting point is the disturbed homeostasis of the organism: neurohormonal, neuromodulatory, and neurotransmission (34).

**Figure 3:**
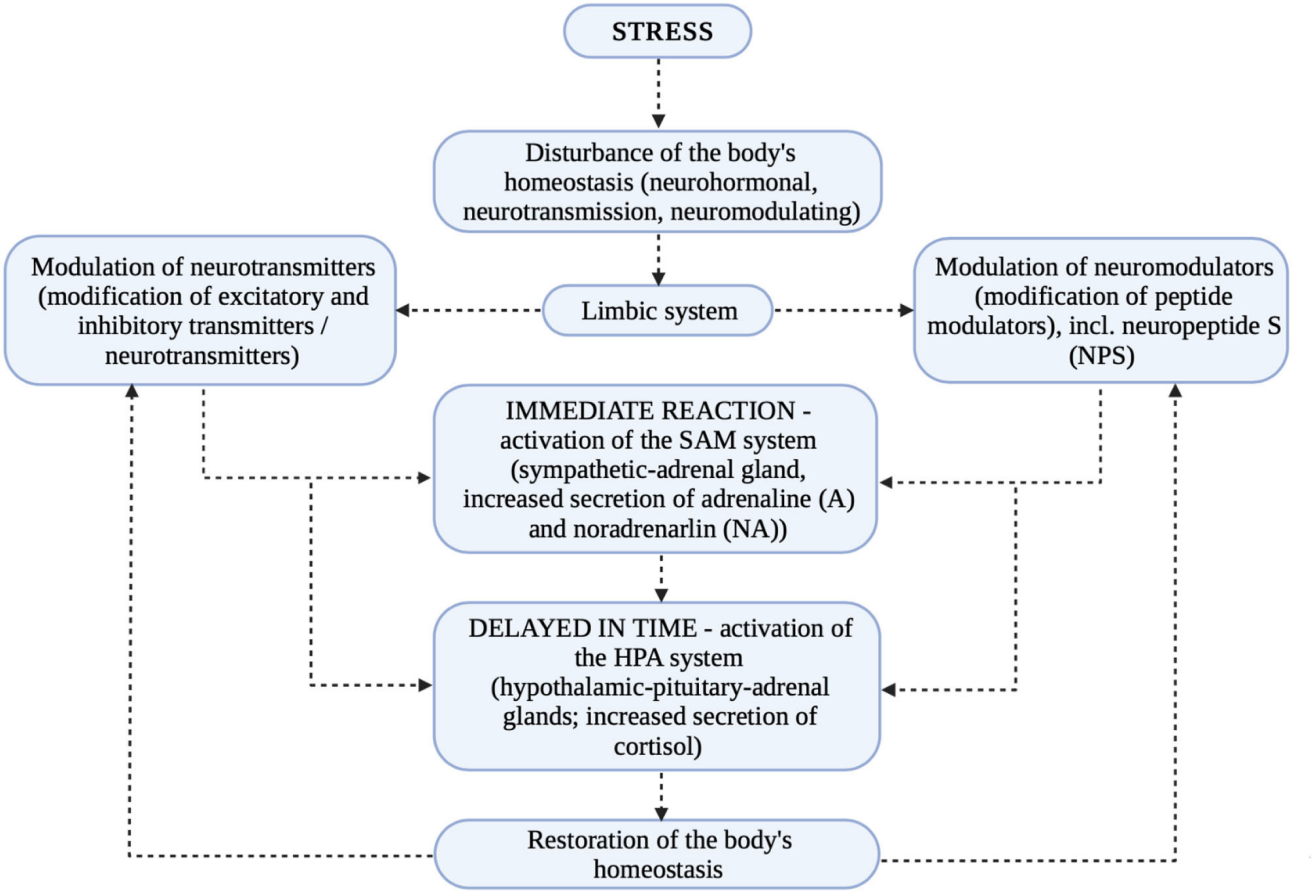
Mechanism of the stress reaction (1–18, 22–25, 29–32, 34, 35).

Thus, since NPS interacts with the HPA axis and is a stress regulator as well as a modulator of endocrine homeostasis it is reasonable to assume that its influence on stabilizing the internal environment is relevant. Since so, its influence on the occurrence of psychiatric disorders is also leading. Typically, disturbances in self-regulatory biological processes result in a range of disturbances in not only interpersonal or social functioning, but also in cognitive functioning (1, 34, 35). Figure 3 presents the stress response and the modulation of neurotransmitters and neuromodulators.

## Neuropeptides and Mental Illness

The involvement of neuropeptides in the development of psychiatric disorders is due to various mechanisms that occur at the cellular and systemic levels. Involvement at the cellular level occurs when a neuropeptide is directly involved in the development of a disease or when its involvement indirectly affects the neurotransmitter responsible for the occurrence of a particular disease. In this case, the result of the abnormal interaction is the modification of the neuropeptide as well as the modification of the neurotransmitter. However, the cellular mechanism itself associated with the anxiolytic effects of NPS remains unclear (36). Now, it has been proven that NPS, via NPSR on the lateral amygdala projection neurons enhances glutamatergic transmission onto GABAergic neurons of the intercalated cell mass of the amygdala, thereby facilitating the extinction of auditory-cued fear memories (36). Neurons responsible for NPS production can be activated by stress and corticotropin-releasing factor, among other things. Their activation affects regions involved with anxiety such as the basolateral amygdala (27), where presynaptically located NPS receptors (NPSR) modulate glutamate release and anxiety (37). Dine et al. tested in inherently fearful mice whether there are differences in neurotransmission and/or plasticity at ventral CA3-CA1 synapses and confirmed them (38). Considering, the above information NPS may become the main target of thymoleptics and anxiolytics drugs in the future.

Since neuropeptides are co-transmitters for neurotransmitters, their importance in the development of mental illnesses is high. Publications in this area emphasize the negative impact of impaired distribution in diseases such as depression, anxiety disorders, post-traumatic stress disorders (PTSD), schizophrenia, and obsessive-compulsive disorder (OCD). The authors demonstrate an adverse effect of the neuropeptide AVP (arginine-vasopressin peptide), also called antidiuretic hormone (ADH), and oxytocin non-apeptide (OXT) on the regulation of neurotransmission in the CNS (39). Due to the high percentage of patients who do not respond to current pharmacological treatment, finding new therapeutic strategies has become an important trend in neurobiology, and it has been shown that neuropeptides are involved not only in the physiology of stress, but can also have a significant clinical impact (40).

The dysregulation of transmission is an effect of stress, which is the main cause and root for the development of many psychiatric diseases. It has been demonstrated that the occurrence of stress (acute or chronic) results in increased expression of corticotropin-releasing factor (CRF) and corticotropic factor, mainly in the hypothalamus, paraventricular nucleus, and pituitary gland (HPA axis) (41). Increased expression of these mediators' results in behavioral changes which accompany symptoms such as anxiety, cognitive, emotional disorders, aggressive behavior, eating disorders, and immunological disorders (42). Dysregulation of the HPA axis is also confirmed by biochemical analysis: increase in cortisol, increase in CRF concentration in limbic structures, increase in CRF concentration in cerebrospinal fluid (43).

**Figure 4:**
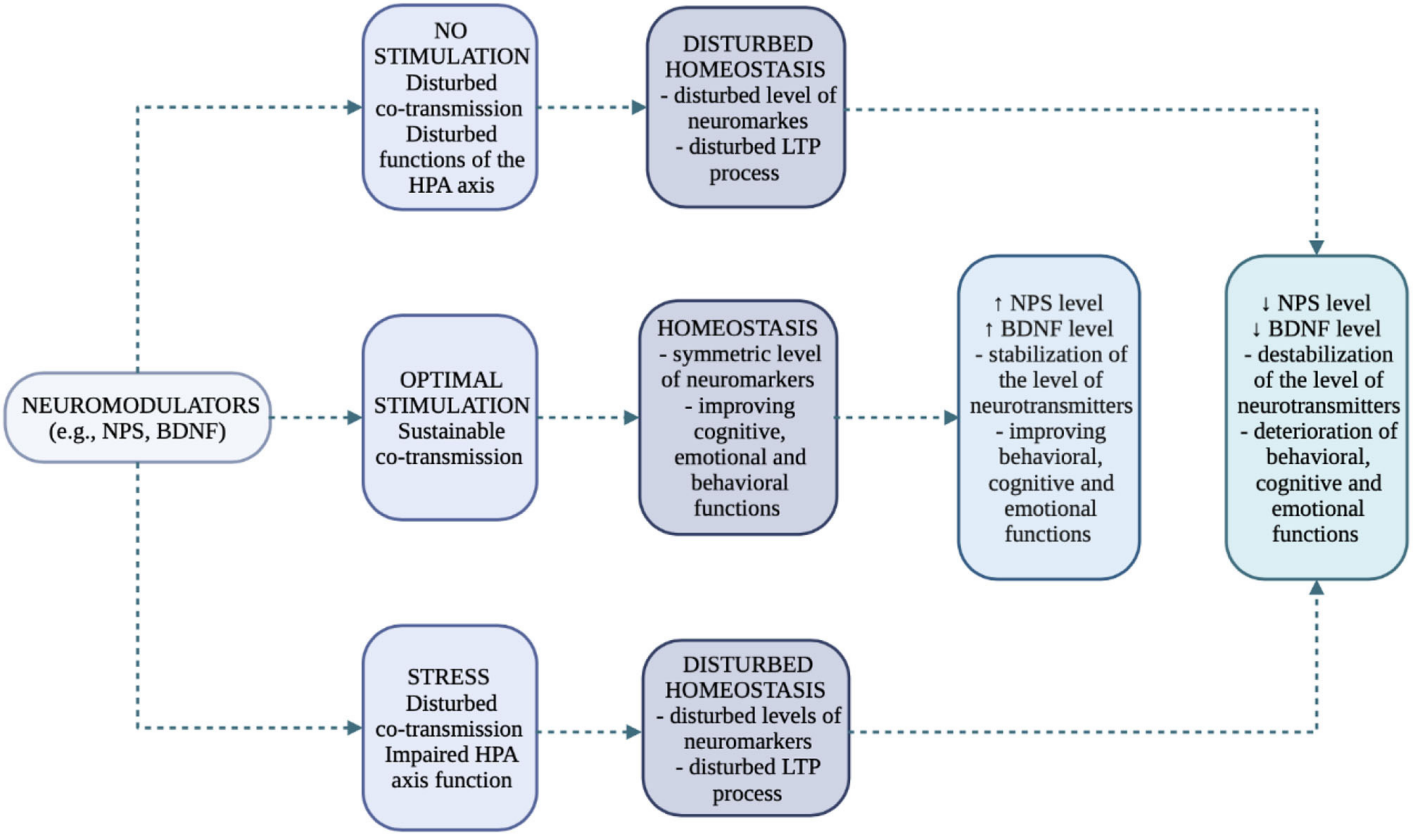
Functions of neuromarkers and their effects in the process of neurogenesis (own work based on the literature) (19, 39, 41–44). The limbic system (cerebral cortex, subcortical structures including the amygdala nuclei, hippocampal bend) is involved in the process of perception of a stressful situation (externally and internally conditioned). A stressful situation has a two-stage course and is subject to primary verification by the amygdala and secondary verification by the hippocampus. The amygdala transmits single sensory signals (autonomic and endocrine responses), while the hippocampus transmits the processed and analyzed signals that are directed from the thalamus and areas of the cerebral cortex. The goal of a synchronized stress response system is to restore internal homeostasis. No Stimulation: decreased level of stimulation; Optimal Stimulation: appropriate regulation; Stress: increased level of stimulation; Disturbed Homeostsis: unbalanced homeostasis; both the deficiency and the excess of stimulation cause disturbed homeostasis in the organism.

All the mentioned dysfunctions are confirmed by Hatzinger (44), who ascribes great importance to neuropeptides, neurotransmitters and additionally neurotrophins (e.g., BDNF - brain derived neurotrophic factor) in the control of HPA axis under stress. Neuropeptides should be particularly emphasized as, in addition to their remote effects, they also function as local modulators of biochemical changes, as they, like neurotransmitters, are released under the influence of cell activation (co-transmission/co-release process) (18, 19). Regarding the NPS/NPSR1 system and the behavioral, emotional, and stress-related changes that occur, we have clinical and preclinical evidence that suggests its importance. However, there is no comprehensive description of the role of NPS/NPSR1 system at important stress points in rodents, and especially humans (45). Model of neurotransmitters/neuromodulators and their potential, final effects in different variants are presented in Figure 4.

## Discussion

Stress response is a defense mechanism of the body, under the influence of which several changes in the distribution of neurotransmitters, neuropeptides, and neurotrophins occur. In the regulation of stress, a great importance is attributed to various systems, but of particular importance is the glutamatergic system (glutamic acid, receptor: N-methyl-d-aspartate—NMDA, and α-amino-3-hydroxy-5-methyl-4-isoxazolepropionic acid - AMPA), which is responsible for processes related to brain neuroplasticity. Brain plasticity refers to its potential to change on both structural and functional level. These changes are accompanied by modification of the number of synapses, in which various mediators are involved. In the normal functioning of CNS, glutametergic system is responsible for synaptic plasticity; its function is to regulate calcium influx into the neuron. This influx is triggered by the cascade of glutamine receptor signals on postsynaptic neurons, which imply the induction of long-term potentiation (LTP) (46, 47), necessary for the consolidation of connections between neurons. The ability to make a network of connections between neurons are activated by NMDA receptor which plays an important role not only in cognitive processes but also in the regulation of the level of neurotransmitters (46).

As mentioned earlier, neuropeptides, including NPS, are co-transmitters for neurotransmitters. They perform functions at both the cellular (signaling molecules) and integrative levels that enable synergistic modulation of brain activity, including enhancement of cognitive and behavioral functions. A key role in neuroplasticity-related processes is played by the earlier mentioned NMDA receptors and BDNF (48).

BDNF is a polypeptide, a growth factor for neurons. Its effect involves regulation of physiological functions related to learning and memory processes, neuro-regenerative functions, and development of serotonergic, cholinergic, noradrenergic, and dopaminergic neurons (49). Its highest expression is found in the hippocampus and cerebral cortex, which supports the idea that this factor is involved in the interaction of the human body with the environment (50–52).

Bearing in mind that various systems are involved in the pathogenesis of psychiatric diseases: dopaminergic, glutamatergic, GABAergic, serotonergic mainly in the hippocampus and frontal lobes, the neurotrophic factor BDNF plays a key role in brain adaptation processes. Its reduced levels in areas of the cerebral cortex and hippocampus were noted in post-mortem studies in patients diagnosed with schizophrenia. As shown in the analyses, the subjects had a lower number of TrkB (brain-derived neurotrophic factor receptor) receptors, which are the primary molecular target for BDNF. Neuronal atrophy and increased apoptosis were also observed (49, 53).

Restoration of the BDNF-dependent adaptive capacity of the brain to environmental conditions is contingent on stimulation, which is a condition of an increased inter-hemispheric transmission of information, consistent with the so-called stimulus effect. Increase of this transmission results in modulation and expression of genes and proteins, which at the molecular level is induced in hippocampal cells by long-term synaptic potentiation (LTP), dependent on glutamatergic AMPA (α-amino-3-hydroxy-5-methyl-4-isoxazolopropionic acid) and NMDA (N-methyl-D-aspartic acid) receptors (54–56).

As mentioned earlier neuropeptides as co-transmitters fulfill various functions in the body depending on the biochemical structure. All of them are based on cooperation, the main goal of which is to maintain the organism's homeostasis (4).

One of them is the group of pituitary peptides which includes vasopressin, oxytocin, and adrenocorticotropic hormone. The action of oxytocin and vasopressin at the level of the CNS is related to the regulation of emotional and cognitive processes. Vasopressin increases the level of anxiety, aggression, and stress, while oxytocin lowers these parameters. The NPS which acts as the coordinator of the HPA axis (57), also engages in the regulatory interaction of social functioning. NPS, as part of the excitatory signaling system, is responsible for many functions, including initiation of arousal and regulation of wakefulness (12, 20, 58), reduction of anxiety (12, 20, 58, 59), promotion of learning and memory enhancement (23, 49), reduction of psychotic functioning (58), regulation of food intake (12, 58), and stimulation of stress hormone release (12, 20, 60, 84). Neuropeptide Y (NPY) is a pancreatic peptide and is one of the most important molecules in the autonomic, central, and peripheral nervous system. In CNS it is located within the cerebral cortex and the limbic system. It coexists with norepinephrine in the neurons of the sympathetic nervous system. Its function in associated with the stimulation of food intake, resistance to stress and vasoconstriction. As demonstrated by Sliwińska et al. (61) and Holzer et al. (62) there is a mutual correlation between the increase in NPY mRNA expression and the stimulation of the HPA axis under stress. Additionally, NPY has similar functions to NPS, it is involved in the control of vasoconstriction, blood pressure, atherosclerotic processes, and intestinal peristalsis and endocrine epithelial function of the gastrointestinal tract and kidney (63–65).

It seems that the most important role of these neuropeptides is to participate in the modification of synapse structures that occurs during LTP. The local transformation of synapses occurring under the influence of LTP depends on the formation of local protein clusters. The result of their action, among others, in the formation of new dendritic spikes and change in the density of glutamate receptors (AMPA, NMDA) responsible for the LTP process. The potential to form new dendrites determines the development of the neuronal network, resulting in the acquisition and consolidation of skills, improvement of cognitive, emotional, and behavioral functions (66).

Neuropeptides, including NPS, as co-transmitters of classical neurotransmitters take part in strengthening or weakening of stimuli in the nervous system, regulating complex processes such as learning, or behavioral processes such as reproduction. Balanced levels of all mediators (an organism's homeostasis) are a necessary condition for developmental processes, including neurogenesis. In case of a stressful situation, in which neurogenesis is inhibited under the influence of glucocorticoids, the activity of BDNF or NPS is incorrectly regulated (67). All this causes that as a result the creation of new connections is inhibited (68–70).

These disorders are greatly influenced by stress which can trigger a chain of changes in oxidative reactions and cause the formation of the free radicals. It should be emphasized that their production also takes place during normal physiological processes but in these situations, it remains under the control of organism, both enzymatic and non-enzymatic (defense mechanisms) (71). In normal reactions of the organism, free radicals play important functions for the cell i.e., proliferation, growth and differentiation of cells and apoptosis. In situation where the balance of control mechanisms is disturbed under the influence of various unfavorable factors including stress, the level of free radicals is also disturbed. Their significant increase leads to damage to the cells and tissues of various organs. It should be added that neurons are the most vulnerable to free radicals' damage because the brain tissue uses more oxygen compared to other organs in the process of obtaining energy (72). In addition, the brain also has a lower activity of antioxidant enzymes: CAT (catalase) and GPx (glutathione peroxidase) and an easier ability to store metals (iron, copper) (73).

The interesting correlation of BDNF and NPS biomarkers requires special emphasis and undoubtedly conducting further research. Their interaction for example an increase in the level of NPS as a marker of anxiety intensity and increased synthesis of BDNF is an intriguing relationship (74, 75). Perhaps it results from the dependence that experiencing and repeated stressful situations are a fixed method of individual reaction, based on the LTP process (76–78). Presumably, constantly reacting with anxiety and the inability to reduce it can lead to excess free radicals and this in turn may result in neurodegenerative disorders (79).

This hypothesis is supported by a study by Cohen et al. in an animal model (Sprague-Dawley rat) which may provide a starting point for future research in humans (80). The author in pioneering analyses, attempted to demonstrate the relationship between the magnitude of stress-related behavioral responses and neuropeptide levels in selected brain areas. The produce of the study consisted in subjecting animals to stress factors (predator odor) for a period of 15 min, and then after 7 days re-analyzing these responses this time induced by acoustic fear-related responses and additional administration of NPY agonist (5 and 10 mg/kg). Established criteria classified animals in terms of their fear responses and in terms of the results obtained in immunohistochemical tests (neuropeptide and BDNF factor expression levels). Animals whose behavior was extremely disturbed showed decreased levels of neuropeptide (NPY) mainly in the hippocampus and the amygdala nucleus and decreased BDNF expression compared to animals whose behavior was minimally disturbed. This result gives rise to the conclusion that NPY and BDNF exhibit endogenous protective effects against stress mainly evident after additional NPY administration. Considering the neuroprotective effect of neuropeptides (NPY, NPS) and neuromodulatory effect of BDNF factor, it can be assumed that the level of stress resistance depends on the level of neuropeptides. The relationship described in the animal model suggests a possible correlation between molecular processes and psychopathological processes in humans. Although the mechanism of interdependence of neuropeptides (mainly NPY) is not fully understood, it is presumed that acting on both pre- and post- synaptic receptors, they influence BDNF expression depending on the type of external stimuli. Given that BDNF exhibits the ability to stimulate its own release this likely guarantees its continued and regenerative signaling at synaptic sites (81). As shown, neuroproliferative interdependencies are confirmed in animal models so further studies in humans are essential to validate these assumptions.

In summary, NPS can be an indicator of body homeostasis, an influence on activity in neurons, an indicator of various mental states: anxiety, restlessness, mood, aggression, and a potential marker of the level of increase of anxiety in mental disorders. Based on the above it can be assumed that in the biological conditioning of mental disorders, NPS will be of great importance not only as a co-transmitter but may prove to be extremely helpful in diagnostics.

## Author Contributions

AM-G and RM: conceptualization. AM-G, RM, JB, and BD: investigation. AM-G and RM: writing—original draft presentation. PK, JB, and RM: writing—review and editing. AM-G and PK: visualization. RM and BD: supervision. AM-G: project administration. All authors have read and agreed to the published version of the manuscript.

## Conflict of Interest

The authors declare that the research was conducted in the absence of any commercial or financial relationships that could be construed as a potential conflict of interest.

## Publisher's Note

All claims expressed in this article are solely those of the authors and do not necessarily represent those of their affiliated organizations, or those of the publisher, the editors and the reviewers. Any product that may be evaluated in this article, or claim that may be made by its manufacturer, is not guaranteed or endorsed by the publisher.

## References

[1] Landowski J. Neurobiology of stress. Neuropsychiatr Neuropsychol Neuropsychiatr Neuropsychol. (2007) 2:26–36.

[2] Kinalski R. Neurofizjologia kliniczna dla neurorehabilitacji. Wydawnictwo MedPharm Polska, Wrocław (2008).

[3] Gunnar M Vazquez D. Stress neurobiology and developmental psychopathology. Dev Psychopathol. (2015) 2:533–577. 10.1002/9780470939390.ch13

[4] Gołyszny M. Stare i nowe neuropeptydy jako modulatory czynności osi stresu (podwzgórze-przysadka nadnercza). Varia Medica. (2018) 2:409–22. Available online at: https://journals.viamedica.pl/varia_medica/article/view/61869/46857

[5] Ground T Goyon S Li Y Eliava M Liu H Charlet A. Neuropeptide S activates paraventricular oxytocin neurons to induce anxiolysis. J Neurosci. (2017) 37:12214–25. 10.1523/JNEUROSCI.2161-17.2017 29118105 PMC6596824

[6] Tafet G Bernardini R. Psychoneuroendocrinological links between chronic stress and depression. Prog Neuropsychopharmacol Biol Psychiatry. (2003) 27:893–903. 10.1016/S0278-5846(03)00162-3 14499305

[7] Gunnar M Quevedo K. The neurobiology of stress and development. Annu Rev Psychol. (2007) 58:145–73. 10.1146/annurev.psych.58.110405.085605 16903808

[8] Windle RJ Shanks N Lightman SL. Central oxytocin administration reduces stress-induced corticosterone release and anxiety behavior in rats. Endocrinology. (1997) 138:2829–34. 10.1210/endo.138.7.5255 9202224

[9] Ciriello J Rosas-Arellano M Solano-Flores LP. Identification of neurons containing orexin-B (hypocretin-2) immunoreactivity in limbic structures. Brain Res. (2003) 967:123–31. 10.1016/S0006-8993(02)04233-6 12650973

[10] Goebel M Stengel A Wang L Lambrecht NWG Tache Y. Nesfatin-1 immunoreactivity in rat brain and spinal cord autonomic nuclei. Neurosci Lett. (2009) 452:241–6. 10.1016/j.neulet.2009.01.064 19348732 PMC2674947

[11] Ryan P Ma S Olucha-Bordonau F Gundlach AL. Nucleus incertus-an emerging modulatory role in arousal, stress, and memory. Neurosci Biobehav Rev. (2011) 35:1326–41. 10.1016/j.neubiorev.2011.02.004 21329721

[12] Xu YL Reinscheid R Huitron-Resendiz S Clark S Wang Z Lin SH. Neuropeptide S: a neuropeptide promoting arousal and anxiolytic-like effects. Neuron. (2004) 43:487–97. 10.1016/j.neuron.2004.08.005 15312648

[13] Rizzi A Vergura R Marzola G Ruzza C Guerrini R Salvadori S. Neuropeptide S is a stimulatory anxiolytic agent: a behavioral study in mice. Br J Pharmacol. (2009) 154:471–9. 10.1038/bjp.2008.96 18376418 PMC2442439

[14] Ionescu I Dine J Yen Y Buell D Herrmann L Holsboer F. Intranasally administered neuropeptide S (NPS) exerts anxiolytic effects following internalization into NPS receptor-expressing neurons. Neuropsychopharmacology. (2012) 37:1323–37. 10.1038/npp.2011.317 22278093 PMC3327839

[15] Adori C Barde S Bogdanovic N Uhlen M Reinscheid R Kovacs G. Neuropeptide S and Neuropeptide S receptor-expressing neuron populations in the human pons. Front Neuroanat. (2015) 9:126. 10.3389/fnana.2015.00126 26441556 PMC4585187

[16] Reinscheid R Xu YL Civelli O. Neuropeptyde S: a new player in the modulation of arousal and anxiety. Mol Interv. (2005) 5:42–6. 10.1124/mi5.1.8 15731503

[17] Herman JP Ostrander MM Mueller NK. Limbic system mechanisms of stress regulation: hypothalamo-pituitary-adrenocortical axis. Prog Neuropsychopharmacol Biol Psychiatry. (2005) 29:1201–13. 10.1016/j.pnpbp.2005.08.006 16271821

[18] Belzung C Yalcin I Griebel G Surget A Leman S. Neuropeptides in psychiatric diseases: an over with a particular focus on depression and anxiety disorders. CNS Neurol Disord Drug Targets. (2006) 5:135–45. 10.2174/187152706776359682 16611088

[19] Erdmann F Kügler S Blaesse P Lange M Skryabin B Pape H. Neuronal expression of the human neuropeptide S receptor NPSR1 identifies NPS-induced calcium signaling pathways. PLoS ONE. (2015) 10:e0117319. 10.1371/journal.pone.0117319 25714705 PMC4340626

[20] Reinscheid RK Ruzza C. Pharmacology, physiology, and genetics of the neuropeptide S system. Pharmaceuticals. (2021) 14:401. 10.3390/ph14050401 33922620 PMC8146834

[21] Xu YL Gall CM Jackson VR Cibelli O Reinscheid RK. Distribution of neuropeptide S receptor mRNA and neurochemical characteristics of neuropeptide S-expressing neurons in the rat brain. J Comp Neurol. (2007) 500:84–102. 10.1002/cne.21159 17099900

[22] Pulkkinen V Majuri M Wang G Holopainen P Obase Y Vendelin J. Neuropeptide S and G protein-coupled receptor 154 modulate macrophage immune responses. Hum Mol Genet. (2006) 15:1667–79. 10.1093/hmg/ddl090 16600990

[23] Okamura N Garau C Duangdao D Clark S Jüngling K Pape H. Neuropeptide S enhances memory during the consolidation phase and interacts with noradrenergic systems in the brain. Neuropsychopharmacology. (2010) 36:744–52. 10.1038/npp.2010.207 21150909 PMC3037424

[24] Permoda-Osip A Rybakowski J. Glutamatergic conception of mood disorders. Psychiatr Pol. (2011) 45:875–88. Available online at: https://pubmed.ncbi.nlm.nih.gov/22335130/22335130

[25] Pittenger C Duman R. Stress, depression, and neuroplasticity: a convergence of mechanisms. Neuropsychopharmacol. (2008) 33:88–109. 10.1038/sj.npp.1301574 17851537

[26] Liu X Zeng J Zhou A Theodorsson E Fabrenkrug J Reinscheid RK. Molecular fingerprint of neuropeptide S – producing neurons in the mouse brain. J Comp Neurol. (2011) 519:1847–66. 10.1002/cne.22603 21452230

[27] Clark SD Duangdao DM Schulz S Zhang L Liu X Xu YL. Anatomical characterization of the neuropeptide S system in the mouse brain by in situ hybridization and immunohistochemistry. J Comp Neurol. (2011) 519:1867–93. 10.1002/cne.22606 21452235

[28] Adori C Barde S Vas S Ebner K Su J Svensson C. Exploring the role of neuropeptide S in the regulation of arousal: a functional anatomical study. Brain Struct Funct. (2016) 221:3521–46. 10.1007/s00429-015-1117-5 26462664

[29] Botticelli L Di Bonavertura E Ubaldi M Ciccocioppo R Cifani C Di Bonavertura MVM. The neural network of neuropeptide S (NPS): implications in food intake and gastrointestinal functions. Pharmaceuticals. (2021) 14:293. 10.3390/ph14040293 33810221 PMC8065993

[30] Kumsta R Chen FS Pape H-S Heinrichs M. Neuropeptide S receptor gene is associated with cortisol responses to social stress in humans. Biol Psychol. (2013) 93:304–7. 10.1016/j.biopsycho.2013.02.018 23466585

[31] Streit F Akdeniz C Haddad L Kumsta R Entringer S Frank J. Sex-specific association between functional neuropeptide S receptor gene (NPSR1) variants and cortisol and central stress responses. Psychoneuroendocrinology. (2017) 76:49–56. 10.1016/j.psyneuen.2016.10.027 27883964

[32] Smith KL Patterson M Dhillo WS Patel SR Semjonous NM Gardiner JV. Neuropeptide S stimulates the hypothalamo-pituitary-adrenal axis and inhibits food intake. Endocrinology. (2006) 147:3510–8. 10.1210/en.2005-1280 16574794

[33] Ebner K Rjabokon A Pape H Singewal N. Increased *in vivo* release of neuropeptide S in the amygdala of freely moving rats after local depolarization and emotional stress. Amino Acids. (2011) 41:991–6. 10.1007/s00726-011-1058-0 21861171 PMC3172411

[34] Markiewicz R. Zastosowanie EEG Biofeedback/Neurofeedback w rehabilitacji psychiatrycznej. Psychiatr Pol. (2017) 51:1095–106. 10.12740/PP/68919 29432505

[35] Iob E Hirschbaum C Steptoe A. Persistent depressive symptoms, HPA-axis hyperactivity, and inflammation: the tole of cognitive and somatic symptoms. Mol Psychiatry. (2020) 25:1130–40. 10.1038/s41380-019-0501-6 31435001 PMC7192852

[36] Cohen H Vainter E Kaplan Z Zohar J Mathe AA. Neuropeptide S in the basolateral amygdala mediates an adaptive behavioral stress response in a rat model of posttraumatic stress disorder by increasing the expression of BDNF and the neuropeptide YY1 receptor. Eur Neuropsychopharmacol. (2018) 28:159–70. 10.1016/j.euroneuro.2017.11.006 29157796

[37] Jungling K Seidenbecher T Sosulina L Lesting J Sangha S Clark SD. Neuropeptide S-mediated control of fear expression and extinction: role of intercalated GABAergic neurons in the amygdala. Neuron. (2008) 59:298–310. 10.1016/j.neuron.2008.07.002 18667157 PMC2610688

[38] Dine J Ionescu IA Avrabos C Yen Y-C Holsboer F Landgraf R. Intranasally applied neuropeptide S shifts a high-anxiety electrophysiological endophenotype in the ventral hippocampus towards a “normal” – anxiety one. PLoS ONE. (2015) 10:e0120272. 10.1371/journal.pone.0120272 25830625 PMC4382147

[39] Soloff M. Regulation of oxytocin action at the receptor level. Life Sci. (1979) 25:1453–60 10.1016/0024-3205(79)90370-9 229376

[40] Rana T Behl T Sehgal A Singh S Sharma N Abdeen N. Exploring the role of neuropeptides in depression and anxiety. Prog Neuro Psychopharmacol Biol Psychiatry. (2022) 114:110478. 10.1016/j.pnpbp.2021.110478 34801611

[41] Givalas L Marmigere F Rage F Ixart G Arancibia S Tapia-Arancibias L. Immobilization stress rapidly and differentially modulates BDNF and TrkB mRNA expression in the pituitary gland of adult male rats. Neuroendocrinology. (2001) 74:148–59. 10.1159/000054681 11528216

[42] Tognoli C Rossi F Di Cola F Baj G Tongiorgi E Terova G. Acute stress alters transcript expression pattern and reduces processing of pro BDNF to mature BDNF in Dicentrarchus labrax. BMC Neurosci. (2010) 11:4. 10.1186/1471-2202-11-4 20074340 PMC2829032

[43] Ai-Min Bao Swaab DF. Corticotropin-releasing hormone and arginine-vasopressin in depression: focus on the human postmortem hypothalmus. Vitam Horm. (2010) 82:338–65. 10.1016/S0083-6729(10)82018-7 20472147

[44] Hatzinger M. Neuropeptides and the Hypothalamic-Pituitary-Adrenocortical (HPA) system: review of recent research strategies in depression. World J Biol Psychiatry. (2009) 1:105–11. 10.3109/15622970009150573 12607206

[45] Tobinski A-M Rappeneau V. Role of the neuropeptide S system in emotionality, stress responsiveness and addiction-like behaviors in rodents: relevance to stress-related disorders. Pharmaceuticals. (2021) 14:780. 10.3390/ph14080780 34451877 PMC8400992

[46] Kazubski W Doroszewska J. Apoptoza w chorobach ośrodkowego układu nerwowego. Wydawnictwo Czelej, Lublin (2008).

[47] Maren S. Synaptic mechanisms of associative memory in the amygdala. Neuron. (2005) 47:783–6. 10.1016/j.neuron.2005.08.009 16157273

[48] Ninan I Bath K Dagar K Perez-Castro R Plummer M Lee F. The BDNF Val66Met polymorphism impairs NMDA receptor-dependent synaptic plasticity in the hippocampus. J. Neurons. (2010) 30:8866–70. 10.1523/JNEUROSCI.1405-10.2010 20592208 PMC2911131

[49] Angelucci F Brene S Math A. BDNF in schizophrenia, depression, and corresponding animal models. Mol Psychiatr. (2005) 10:345–52. 10.1038/sj.mp.4001637 15655562

[50] Tyler W Alonso M Bramham C Pozzo-Miller L. From acquisition to consolidation: on the role of brain-derived neurotrophic factor signaling in hippocampal-dependent learning. Learn Mem. (2002) 9:224–37. 10.1101/lm.51202 12359832 PMC2806479

[51] Siegelbaum S Kandel E. Learning-related synaptic plasticity. Curr Opin Neurobiol. (1991) 1:113–20. 10.1016/0959-4388(91)90018-3 1822291

[52] Malenka R Baer M. LTP and LTD: an embarrassment of riches. Neuron. (2004) 44:5–21. 10.1016/j.neuron.2004.09.012 15450156

[53] Libman-Sokołowska M Drozdowicz E Nasierowski T. BDNF as a biomarker in the course and treatment of schizophrenia. Psychiatr Pol. (2015) 49:1149–58. 10.12740/PP/37705 26909392

[54] Purves D Augustine G Fitzpatrick D. Neuroscience. Sinauer Associates, Inc. (2004).

[55] Fink C Meyer T. Molecular mechanisms of CaMKII activation in neuronal plasticity. Curr Opin Neurobiol. (2002) 12:293–9. 10.1016/S0959-4388(02)00327-6 12049936

[56] Risher W Eroglu C. Thrombospondins as key regulators of synaptogenesis in the central nervous system. Matrix Biol. (2012) 31:170–7. 10.1016/j.matbio.2012.01.004 22285841 PMC3961754

[57] Wójciak P Remlinger-Molenda A Rybakowski J. The role of oxytoxin and vasopressin in central nervous system activity and mental disorders. Psychiatr Pol. (2012) 46:1043–52. Available online at: https://pubmed.ncbi.nlm.nih.gov/23479945/23479945

[58] Donner J Haapakoski R Ezer S Melen S Pirkola S Gratacòs M. Assessment of Neuropeptide S system in anxiety disorders. Biol Psychiatry. (2010) 68:474–83. 10.1016/j.biopsych.2010.05.039 20705147

[59] Zoicas I Menon R Neumann I. Neuropeptide S reduces fear and avoidance of conspecifics induced by social fear conditioning and social defeat, respectively. Neuropharmacology. (2016) 108:284–91. 10.1016/j.neuropharm.2016.03.054 27044664

[60] Tilbrook AJ Clarke IJ. Neuroendocrine mechanisms of innate states of attenuated responsiveness of hypotkalmo-pituitary adrenal axis to stress. Front Neuroendocrinol. (2006) 27:285–307. 10.1016/j.yfrne.2006.06.002 16930683

[61] Sliwińska-Mossoń M Borowiecka K Milenerowicz H. Neuropeptides Y, YY, PP and their clinical significance. Postepy Hig Med Dosw. (2013) 18:631–6. 10.5604/17322693.1058890 24018426

[62] Holzer P Reichmann F Farzi A. Neuropeptide Y, peptide YY and pancreatic polypeptide in the gut-brain axis. Neuropeptides. (2012) 46:261–74. 10.1016/j.npep.2012.08.005 22979996 PMC3516703

[63] Peng Y Han R Chang M Zhang L Zhang R Li W. Central Neuropeptide S inhibits food intake in mice through activation of Neuropeptide S receptor. Peptides. (2010) 31:2259–63. 10.1016/j.peptides.2010.08.015 20800637

[64] Britton K Akwa Y Spina M Koob GF. Neuropeptide Y blocks anxiogenic-like behavioral action of corticotropin-releasing factor in an operant conflict test and elevated plus maze. Peptides. (2000) 21:37–44. 10.1016/S0196-9781(99)00169-2 10704717

[65] Giesbrecht C Mackay J Silveira H Urban J Colmers W. Countervailing modulation of in by neuropeptide Y and corticotrophin-releasing factor in basolateral amygdala as a possible mechanism for their effects on stress-related behaviors. J Neurosci. (2010) 30:16970–82. 10.1523/JNEUROSCI.2306-10.2010 21159967 PMC3432911

[66] Markiewicz R Masiak J. Evaluation of cognitive deficits in schizophrenia using event-related potentials and rehabilitation influences using EEG Biofeedback in patients diagnosed with schizophrenia. Psychiatr Pol. (2019) 53:1261–73. 10.12740/PP/OnlineFirst/102622 32017816

[67] Kawashima H Numakawa T Kumamaru E Adachi N Mizuno H Ninomiya M. Glucocrticoid attenuated brain-derived neurotrophic factor-dependent upregulation of glutamate receptors via the suppression of microRNA-132 expression. Neuroscience. (2010) 165:1301–11. 10.1016/j.neuroscience.2009.11.057 19958814

[68] Liu DY Shen XM Yuan FF Guo OY Zhong Y Chen JG. The physiology of BDNF and its relationship with ADHD. Mol Neurobiol. (2015) 52:1467–76. 10.1007/s12035-014-8956-6 25354496

[69] Suri D Vaidya VA. Glucocorticoid regulation of brain-derived neurotrophic factor: relevance to hippocampal structural and functional plasticity. Neuroscience. (2013) 3:196–213. 10.1016/j.neuroscience.2012.08.065 22967840

[70] Radley J Sisti H Rocher H McCall T Hof PR McEwen BS. Chronic behavioral stress induces apical dendritic reorganization in pyramidal neurons of the medial prefrontal cortex. Neuroscience. (2004) 125:1–6. 10.1016/j.neuroscience.2004.01.006 15051139

[71] Sheu SS Nauduri D Anders MW. Targeting antioxidans to midochondria: a new therapeutic direction. Biochim Biophys Acta. (2006) 1762:256–65. 10.1016/j.bbadis.2005.10.007 16352423

[72] Halliwell B. Oxidative stress and neurodegeneration: where are we now? J Neurochem. (2006) 97:1634–58. 10.1111/j.1471-4159.2006.03907.x 16805774

[73] Hainwell B. Role of free radicals in the neurodegenerative diseases: therapeutic implications for antioxidant treatment. Drugs Aging. (2001) 18:685–716. 10.2165/00002512-200118090-00004 11599635

[74] Smith JP Achua JK Summers TR Ronan PJ Summers CH. Neuropeptide S and BDNF gene expression in the amygdala are influenced by social decision-making under stress. Front Behav Neurosci. (2014) 8:121. 10.3389/fnbeh.2014.00121 24782729 PMC3986560

[75] Markiewicz R Kozioł M Olajossy M Masiak J. Can brain-derived neutrophic factor (BDNF) be an indicator of effective rehabilitation interventions in schizophrenia? Psychiatr Pol. (2018) 52:819–34. 10.12740/PP/OnlineFirst/76040 30584816

[76] Orsini C Maren S. Neural and cellular mechanisms of fear and extinction memory formation. Neurosci Biobehav Rev. (2012) 36:773–1802. 10.1016/j.neubiorev.2011.12.014 22230704 PMC3345303

[77] Cannella N Kallupi M Ruggeri B Ciccocioppo R Ubaldi M. The role of the neuropeptide S system in addiction: focus on its interaction with the CRF and hypocretin/orexin neurotransmission. Prog Neurobiol. (2013) 100:48–59. 10.1016/j.pneurobio.2012.09.005 23041581

[78] Arendt D Smith J Bastida C Prasad M Oliver K Eyster K. Contrasting hippocampal and amygdalar expression of genes related to neural plasticity during escape from social aggression. Physiol Behav. (2012) 107:670–9. 10.1016/j.physbeh.2012.03.005 22450262 PMC4372993

[79] Karpińska A Gromadzka G. Oxidative stress, and natural antioxidant mechanism: the role in neurodegeneration. From molecular mechanism to therapeutic strategies. Postepy Hig Med Dosw. (2013) 67:43–53. 10.5604/17322693.1029530 23475482

[80] Cohen C Zohar J. An animal model of posttraumatic stress disorder: the use of cut-off behavioral criteria. Ann NY Acad Sci. (2004) 1032:167–78. 10.1196/annals.1314.014 15677404

[81] Bramham C Messaoudi E. BDNF function in adult synaptic plasticity: the synaptic consolidation hypothesis. Prog Neurobiol. (2005) 76:99–125. 10.1016/j.pneurobio.2005.06.003 16099088

[82] Bengoetxea X Goedecke L Remmes J Blaesse P Grosch T Lesting J. Human-specific neuropeptide S receptor variants regulate fear extinction in the basal amygdala of male and female mice depending on threat salience. Biol Psychiatry. (2021) 90:145–55. 10.1016/j.biopsych.2021.02.967 33902914

[83] Soares-Silva M Diniz FF Gomes GN Bahia D. The mitogen-activated protein kinase (MAPK) pathway: role in immune evasion by Trypanosomatidis. Front Microbiol. (2016) 7:183. 10.3389/fmicb.2016.00183 26941717 PMC4764696

[84] Okamura N Reinscheid R Ohgake S Iyo M Hashimoto K. Neuropeptide S attenuates neuropathological, neurochemical, and behavioral changes induced by the NMDA receptor antagonist MK-801. Neuropharmacology. (2010) 58:166–72. 10.1016/j.neuropharm.2009.06.027 19576911 PMC2783386

